# Predictors of *Chlamydia trachomatis* Infection Among Women Attending Rural Midwest Family Planning Clinics

**DOI:** 10.1155/S1064744901000023

**Published:** 2001

**Authors:** Tami M. Hilger, Elaine M. Smith, Kevin Ault

**Affiliations:** 1 Department of Epidemiology College of Public Health University of Iowa Iowa City IA USA; 2 Department of Obstetrics and Gynecology College of Medicine University of Iowa Iowa City IA USA; 3 Department of Preventive Medicine 2800 SB College of Medicine Iowa City IA 52242 USA

## Abstract

*Objective:* To determine predictors of *Chlamydia trachomatis* infection among women 14–24 years of age attending family planning clinics throughout a rural Midwestern state.

*Methods:* The study population included 16 756 women between the ages of 14 and 24 years attending family planning clinics for annual examinations throughout the state of Iowa in 1997. All women under 25 years of age having annual exams were tested for *C. trachomatis* during the visit. At the time of exam, both behavioral and demographic data were collected on all women participating in the study.

*Results:* The majority of women in the study (96%) reported no symptoms of chlamydia. Only 2.5% of all women had a positive test result. In the multivariate model, the odds ratios were significantly increased among the youngest age (14–17 years; OR = 2.2), those with mucopurulent cervicitis (OR = 3.4), cervical friability (OR = 2.2), symptomatic for infection (OR = 1.8), risk history (OR = 1.6), and black race (OR = 1.2) and predictive of a *C. trachomatis* infection.

*Conclusions:* Risk factors predictive of *C. trachomatis* infection among younger aged women attending family planning clinics in a Midwest rural population are consistent with predictors of infection among women attending family planning clinics across theUnited States. The overall findings suggest the importance of developing screening guidelines as a means of lowering chlamydia rates. This may be a particularly difficult task in light of the low rate of symptoms that would lead a woman to seek medical care, even in younger age women who are at higher risk. In addition, screening guidelines would be more difficult to implement in a rural setting.


*Infect Dis Obstet Gynecol 2001;9:3–8*

### Predictors of *Chlamydia trachomatis* infection among women attending rural Midwest family planning clinics

**Tami M. Hilger[1], Elaine M. Smith[1,2] and Kevin Ault[2]**

[1]*Department of Epidemiology, College of Public Health, University of Iowa, Iowa City, IA*
[2]*Department of Obstetrics and Gynecology, College of Medicine, University of Iowa, Iowa City, IA*

***Objective:*** To determine predictors of *Chlamydia trachomatis* infection among women 14–24 years of age attending family planning clinics throughout a rural Midwestern state.
***Methods:*** The study population included 16 756 women between the ages of 14 and 24 years attending family planning clinics for annual examinations throughout the state of Iowa in 1997. All women under 25 years of age having annual exams were tested for *C. trachomatis* during the visit. At the time of exam, both behavioral and demographic data were collected on all women participating in the study.
***Results:*** The majority of women in the study (96%) reported no symptoms of chlamydia. Only 2.5% of all women had a positive test result. In the multivariate model, the odds ratios were significantly increased among the youngest age (14–17 years; OR = 2.2), those with mucopurulent cervicitis (OR = 3.4), cervical friability (OR = 2.2), symptomatic for infection (OR = 1.8), risk history (OR = 1.6), and black race (OR = 1.2) and predictive of a *C. trachomatis* infection.
***Conclusions:*** Risk factors predictive of *C. trachomatis* infection among younger aged women attending family planning clinics in a Midwest rural population are consistent with predictors of infection among women attending family planning clinics across the United States. The overall findings suggest the importance of developing screening guidelines as a means of lowering chlamydia rates. This may be a particularly difficult task in light of the low rate of symptoms that would lead a woman to seek medical care, even in younger age women who are at higher risk. In addition, screening guidelines would be more difficult to implement in a rural setting.

Key Words: Sexually Transmitted Disease, Screening, Family Planning

Genital infection resulting from *Chlamydia trachomatis* remains the most prevalent sexually transmitted bacterial infection in the United States today, with an estimated 4 million cases occurring every year[1–7]. Chlamydial infections have been reported as being asymptomatic in up to 70% of infected individuals, and may become problematic when left untreated[3,7,8]. Sequelae that result from untreated *C. trachomatis* infection include pelvic inflammatory disease, ectopic pregnancy, chronic pelvic pain and tubal infertility[5,7,9–14]. In addition, conjunctivitis and pneumonia may result in infants born to mothers infected with *C. trachomatis*[7]. Sequelae of *C. trachomatis* infections can be extremely costly and may develop into further health-threatening complications. In the United States alone, costs related to chlamydia infection have been estimated to reach as much as $2.4 billion annually[15]. As a result, screening programs designed to identify both symptomatic and

Correspondence to: Dr Elaine M. Smith, MPH, PhD, Department of Preventive Medicine, 2800 SB, College of Medicine, Iowa City, IA 52242, USA. Email: elaine-smith@uiowa.edu asymptomatic individuals earlier in the course of infection are crucial to the successful treatment and control of *C. trachomatis* infections.

Much research has examined where screening efforts should be focused. There has been a substantial amount of debate as to whether universal screening or selective screening programs are more effective[4,6,11,13,15,16]. Because the practice of universal screening is extremely costly and may not be necessary in populations where the prevalence of chlamydia infection is low, many programs have developed screening criteria for select populations which rely on targeting those individuals with risk factors known to be predictive of infections. Depending on the target population, some programs appear to be cost-effective in the detection and treatment of infected individuals[13].

The main objective of this study was to examine predictors of *C. trachomatis* infection among women attending family planning clinics throughout the rural state of Iowa. Furthermore, we compared these predictors of chlamydia infection seen in women to those residing in other geographic locations in the United States. Using the results of this study and others, we can suggest populations of sexually active individuals in rural Midwest communities where screening programs should be targeted and whether this population has different risks and health care screening needs to that of other parts of the country.

### SUBJECTS AND METHODS

### Study population

The study population consisted of 16 756 women between the ages of 14 and 24 years who were attending family planning clinics throughout the state of Iowa as part of the Centers for Disease Control's Region VII Infertility Prevention Project. All women included in the study made an annual visit to the clinics between January 1, 1997 and December 31, 1997.

### Data collection

Behavioral and demographic data were collected on all women screened for chlamydia using a standardized form at each annual visit. Clinical signs of infection included cervical friability, mucopurulent cervicitis, cervicitis, and/or pelvic inflammatory disease, and only one of these needed to be present in order for the physician to classify that individual as having clinical signs. As part of the CDC screening project, all clinicians were extensively trained at the beginning of the project and then annually thereafter. The training programs were augmented by a quality assurance program and definitions of clinical characteristics were standardized as part of the training and as described in the project manual.

Cervical friability was defined as easily induced bleeding with the initial swab during culture. Yellow or green mucopurulent discharge from the cervix, with infection, was classified as mucopurulent cervicitis. Cervicitis was defined to include any of the following: (1) edema, erythema or follicle-like lesion in an area of ectopy (the extension of columnar epithelium onto the ectocervix); or (2) cervical mucus with ten or more polymorphonuclear leukocytes per × 1000 microscopic level. Pelvic inflammatory disease was defined as the findings of lower abdominal tenderness, adnexal tenderness, and cervical motion tenderness in a patient complaining of pelvic pain. A women was characterized as having a risk history if any of the following was applicable: (1) multiple sexual partners (two or more within 90 days) and/or sexual partners with multiple partners, (2) new sexual partner since last screening, (3) contact with STD (symptomatic males, or history of sex partner with symptoms of chlamydia); or (4) request a test based on risk history. Whether or not a patient was symptomatic (e.g. pelvic pain or vaginal discharge) was by self-report.

### Laboratory methods

Swab specimens were obtained from the patients during their annual visits and were shipped to the University of Iowa Hygienic Laboratory. Processing occurred at the laboratory using the SYVA Micro Trak II chlamydia EIA test.

### Statistical analysis

Because the CDC screening criteria were applied differently depending upon the age of the patient, data included in this report were analyzed for younger aged women only (ages 14–24 years). The relationship between chlamydia infection and potential risk factors or confounders were examined and a test of association was obtained by the Pearson's $\chi^2$ statistic, the Fischer's Exact two-sided test or the Mantel-Haenszel test as a means of comparing categorical data in Tables 1 and 2. Odds ratios and 95% confidence intervals were generated to determine statistical significance. Where *p*-values were calculated, results were considered to be significant at values equal to or less than 0.05.

Univariate logistic regression analysis using SAS version 6.0 (SAS Institute Inc., Cary, NC) was performed as a means of generating odds ratios to assess the relationship between chlamydia infection and each individual risk factor. Both unadjusted and age-adjusted odds ratios were calculated with their corresponding 95% confidence intervals to determine significant associations at the 5% level. Following univariate logistic regression analysis, multivariate models were generated for both age groups to determine the best predictors of chlamydia infection.

### RESULTS

There were 16 972 different patients aged 24 years of age and younger who were classified as patients attending family planning clinics annually throughout the state of Iowa between January 1, 1997 and December 31, 1997. The mean age of the women was 20.1 years and the racial background was predominantly white (96.2%). Overall, 95.6% of women had no symptoms of an infection for chlamydia and only 2.5% of women in this study tested positive regardless of whether they reported symptoms or not. Among those who were symptomatic for chlamydia, over 7% tested positive compared to only 2% of those without symptoms. A much higher percentage of blacks compared to whites had a positive test.

**Table 1** Characteristics of women 24 years of age and younger attending family planning clinics throughout the State of Iowa ($n$ = 16 972)

| *Variable* | *Category* | *Total number* | *Number positive* | *% Positive* | *p-value* |
|---|---|---|---|---|---|
| Age group (years) | 14–17 | 2577 | 106 | 4.1 | 0.001 |
| | 18–21 | 9124 | 218 | 2.4 | |
| | 22–24 | 5040 | 96 | 1.9 | |
| Clinical signs | Yes | 1091 | 69 | 6.3 | 0.001 |
| | No | 15 015 | 341 | 2.3 | |
| Cervicitis | Yes | 166 | 11 | 6.6 | < 0.001* |
| | No | 16 806 | 416 | 2.5 | |
| Mucopurulent cervicitis | Yes | 159 | 22 | 13.8 | < 0.001* |
| | No | 16 183 | 405 | 2.4 | |
| Cervical friability | Yes | 789 | 48 | 6.1 | 0.001 |
| | No | 16 813 | 379 | 2.4 | |
| Risk history | Yes | 7920 | 258 | 3.3 | 0.001 |
| | No | 8716 | 160 | 1.8 | |
| Symptomatic | Yes | 732 | 53 | 7.2 | 0.001 |
| | No | 16 036 | 368 | 2.3 | |
| Race[†] | Asian/Pacific Island | 129 | 4 | 3.1 | 0.001 |
| | Black | 335 | 31 | 9.3 | |
| | Native American/Alaskan | 15 | 1 | 6.7 | |
| | White | 16 330 | 386 | 2.4 | |
| | Unknown | 163 | 5 | 3.1 | |

*$p$-Values are based on Pearson's $\chi^2$ or Fischer's Exact two-sided test when expected counts are less than 25; [†]Referent: Caucasian

As shown in Table 1, 6.3% of the women presented with clinical signs of chlamydia infection. The most commonly reported signs of those who tested positive were mucopurulent cervicitis, cervical friability, and cervicitis. This compares to about only 2.5% of those without any of these clinical signs who subsequently had a positive chlamydia infection. Almost half of the women had a history of sexual activity that placed them at higher risk for chlamydia infection.

Multivariate logistic analyses examined risks of infection, controlling for confounders and other potential risks. Results indicated that in younger aged women the following were predictive of infection (Table 2): (1) the youngest age group (14–17 years *vs* 22–24 years); (2) clinical signs of cervicitis, mucopurulent cervicitis and cervical friability, and (3) patient characteristics of symptomatic, risk history, and race (black). The strongest predictor of chlamydia infection was mucopurulent cervicitis (adjusted OR = 3.4). Cervical friability (OR = 2.2) and young age at diagnosis, age 14–17 years (OR = 2.2), were also associated with an increased risk of chlamydia infection. Those aged 18–21 years did not have a significantly increased risk over those aged 22–24 years. Although the ORs were elevated, the presence of cervicitis did not significantly elevate the risk of chlamydia. Infected women were more likely to be symptomatic (OR = 1.8) than uninfected women and to report a history of risky sexual behavior (OR = 1.6). In addition, African-American women had a significantly higher risk of chlamydia infection compared to white women (OR = 1.2).

**Table 2** Multivariate analysis of risk factors associated with *C. trachomatis* infection in women ≤ 24 years of age

| *Risk factor* | *Odds ratio* | *95% CI* |
|---|---|---|
| Age 14–17 years* | 2.23 | (1.67, 2.98) |
| Age 18–21 years* | 1.24 | (0.97, 1.59) |
| Cervicitis | 3.42 | (0.69, 2.87) |
| Mucopurulent cervicitis | 3.42 | (2.01, 5.80) |
| Cervical friability | 2.16 | (1.55, 3.02) |
| Symptomatic | 1.84 | (1.29, 2.64) |
| Risk history | 1.56 | (1.27, 1.91) |
| Race (Black *vs* White) | 1.24 | (1.05, 1.47) |

*Compared to group aged 22–24 years

### DISCUSSION

The current study examined predictors of infection in a rural Midwestern population of young women screened for chlamydia. The infection rate measured in this population was similar to that reported in other studies, 2.5% compared to 2.7–3.5% of women in family planning and other types of clinical settings[5,12], but lower than in other reports[3,17]. Whether infection rates are different by health care setting in this rural population because access is more difficult to obtain than it is among urban populations is uncertain. At the time of this study there were no active statewide screening programs for sexually transmitted diseases or chlamydia specifically.

A major advantage of this study is that it utilized one of the largest, multi-clinic database sources to assess risks of infection, thus capturing a representative sample of the larger population. The results confirm risk factors for *C. trachomatis* infection found in similar studies of women attending family planning clinics in different geographic locations of the United States. Other studies have also confirmed cervical friability[5,9,11], mucopurulent cervicitis[5,7,11], cervicitis[7,9,13–15], the presence of clinical signs of infection[5,7,9,11,13], whether or not a patient is symptomatic[1], risk history, and race[5,9] as predictive of chlamydia infection.

Results from the multivariate analysis were also consistent with those from other studies[18-20] regarding age, namely that the youngest aged women, 14–17 years, had the highest risk. In contrast, those aged 18–21 years had only a slightly higher probability of infection compared to those aged 22–24 years. Although it did not achieve statistical significance, it would appear that cervicitis was a clinically significant factor with the risk elevated more than three-fold. Other studies also have detected elevated rates of this clinical characteristic in women with chlamydia[21,22].

The analysis was restricted to those women who were classified as presenting for their annual visit to the family planning clinic. The assumption was that they were defined by the clinic record as visiting the clinic one time during the study period for their annual exam. If women had more than this annual visit during the one-year study period, our estimates may be biased depending on their *C. trachomatis* infection status and the risk factors they presented. However, women who were suspected of having chlamydia infection during their annual visit would be followed up in a different clinic status category that was not included in this analysis. Women who might have sought care because of STD symptoms or other reasons, and not for their annual visit were classified differently and also were not included in the annual visit group. Because our findings were generally consistent with those of other studies, including one other study in which patient identifiers were unknown but showed risk factors similar to other reports[6], it is likely that potential misclassification was limited.

One limitation of this study is the lack of racial and ethnic diversity in the study population, since the majority of women residing in the rural state of Iowa identify themselves as 'white'. As a result, some of our findings may not be generalizable to populations with more diverse racial and ethnic backgrounds. Nonetheless, there was a sufficient African-American population to evaluate for risk of infection. As found in other studies[1,5], they appear to be at higher risk of contracting chlamydia. In contrast to this limitation, this population is more characteristic of women for whom access to routine health care is limited by distance to a physician or clinic. It is important to determine the prevalence of chlamydia in this rural population because it is likely to indicate the extent of infection. In addition, it will show health care providers and state and local health departments the problems of providing adequate health care to screen this high risk group.

This study indicates that among women attending family planning clinics in a rural state who are sexually active, present with symptoms or clinical signs, a risky sexual history, younger age, or black race are the most likely to be infected with *C. trachomatis*. In light of these findings, chlamydia control mechanisms should implement screening programs that target these subgroups of individuals. With the high rates of infection, it is extremely important to detect these cases early to reduce the spread and adverse effects of this costly, but preventable, sexually transmitted disease. Patients may not only be asymptomatic but also may be unaware of the implication of their symptoms until they are sufficiently symptomatic to create concern on the woman's part. Studies suggest that selective screening is cost-effective when infection rates are low, as found in this population[13]. Such a program that incorporates chlamydia screening as the standard of care for young, sexually active women, whether in public or private medical care settings, would provide an important method to prevent pelvic inflammatory disease, a major cause of infertility and ectopic pregnancy. The difficulties associated with rural health care among low income women or those who live in an area with limited health care access needs to be a priority of rural health care providers and state governmental funding. Such preventive programs could provide a wide variety of early screening tests simultaneously, while also administering to the health educational needs of inexperienced women and to maintain preventive benefits in this high-risk population. These screening sites also would provide a contact source for continued care and treatment information.

### REFERENCES

1. Burstein GR, Waterfield G, Joffe A, *et al.* Screening for gonorrhea and chlamydia by DNA amplification in adolescents attending middle school health centers – opportunity for early intervention. *Sex Transm Dis* 1998;25:395–402
2. Shaw E, Roberts D, Connor PD. Prevalence of and risk factors for chlamydia in a rural pregnant population. *J Fam Pract* 1995;41:257–60
3. Katz BP, Blythe MJ, Van Der Pol B, *et al.* Declining prevalence of chlamydia infection among adolescent girls. *Sex Transm Dis* 1996; 23:226–9
4. Scholes D, Stergachis A, Heidrich FE, *et al.* Prevention of pelvic inflammatory disease by screening for cervical chlamydia infection. *N Engl J Med* 1996;334:1362–6

5. Gershman KA, Barrow JC. A tale of two sexually transmitted diseases – prevalences and predictors of chlamydia and gonorrhea in women attending Colorado family planning clinics. *Sex Transm Dis* 1996;23:481–7
6. Mertz KJ, Levine WC, Mosure DJ, *et al.* Trends in the prevalence of chlamydia infections – the impact of community-wide testing. *Sex Transm Dis* 1997; 24:169–75
7. Reddy SP, Yeturu BS, Slupik R. *Chlamydia trachomatis* in adolescents: a review. J *Pediatr Adolesc Gynecol* 1997;10:59–72
8. Stokes T. Screening for chlamydia in general practice: a literature review and summary of the evidence. *J Public Health Med* 1997;19:222–32
9. Mosure DJ, Berman S, Kleinbaum D, Halloran ME. Predictors of *Chlamydia trachomatis* infection among female adolescents: a longitudinal analysis. *Am J Epidemiol* 1996;144:997–1003
10. Quinn TC, Gaydos C, Shepherd M, *et al.* Epidemiologic and microbiologic correlates of *Chlamydia trachomatis* infection in sexual partnerships. *JAMA* 1996;276:1737–42
11. Mosure DJ, Berman S, Fine D, *et al.* Genital chlamydia infections in sexually active female adolescents: do we really need to screen everyone? *J Adolesc Health* 1997;20:6–13
12. Jonsson M, Karlsson R, Persson K, *et al.* The influence of sexual and social factors on the risk of *Chlamydia trachomatis* infections: a population-based serologic study. *Sex Transm Dis* 1995;22: 355–63
13. Marrazzo JM, Celum CL, Hillis SD, *et al.* Performance and cost-effectiveness of selective screening criteria for *Chlamydia trachomatis* infection in women – implications for a national chlamydia control strategy. *Sex Transm Dis* 1997; 24:131–40
14. Thompson SE, Washington AE. Epidemiology of sexually transmitted *Chlamydia trachomatis* infections. *Epidemiol Rev* 1983;5:96–123
15. Orr DP, Fortenberry JD. Screening adolescents for sexually transmitted infections. *JAMA* 1998;280: 564–5
16. Hillis S. Screening for chlamydia – a key to the prevention of PID. *N Engl J Med* 1996;34: 1399–400
17. Johnson BA, Poses RM, Fortner AC. Derivation and validation of a clinical diagnostic model for chlamydial cervical infection in university women. *JAMA* 1990;264:3161–5
18. Han Y, Coles FB, Hipp S. Screening criteria for *Chlamydia trachomatis* in family planning clinics: accounting for prevalence and clients' characteristics. *Fam Plan Perspect* 1997;29:163–6
19. Handsfield HH, Jasman LL, Roberts PL, *et al.* Criteria for selective screening for *Chlamydia trachomatis* infection in women attending family planing clinics. *JAMA* 1986;255:1730–4
20. Harrison HR, Costin M, Meder JD, *et al.* Cervical *Chlamydia trachomatis* infection in university women: relationship to history, contraception, ectopy, and cervicitis. *Am J Obstet Gynecol* 1985;151:244–50
21. Addiss DG, Vaughn ML, Ludka D, *et al.* Decreased prevalence of *Chlamydia trachomatis* infection associated with a selective screeing program in family planing clinics in Wisconsin. *Sex Transm Dis* 1993;20:28–35
22. Addiss DG, Vaughn ML, Holzhueter MA, *et al.* Selective screening for *Chlamydia trachomatis* infection in nonurban family planning clinics in Wisconsin. *Fam Plan Perspect* 1987;19:252–6

Received 10/30/00; Accepted 01/22/01
